# Prognostic Value of 2-Deoxy-2-[^18^F]fluoro-D-glucose Positron Emission Tomography/Computed Tomography after Autologous Hematopoietic Stem Cell Transplantation in Lymphoma Using Deauville Scores

**DOI:** 10.1155/2021/5510825

**Published:** 2021-04-15

**Authors:** Na Dai, Yeye Zhou, Shengming Deng, Shibiao Sang, Yiwei Wu

**Affiliations:** Department of Nuclear Medicine, The First Affiliated Hospital of Soochow University, Suzhou, Jiangsu 215006, China

## Abstract

**Purpose:**

In the present study, we mainly aimed to evaluate the prognostic value of 2-deoxy-2-[^18^F]fluoro-D-glucose ([^18^F]F-FDG) positron emission tomography (PET)/computed tomography (CT) after autologous stem cell transplantation (ASCT) in lymphoma. *Procedures*. A total of 76 lymphoma patients who benefited from [^18^F]F-FDG PET-CT (within 3 months and 3–6 months) after ASCT in our institution between April 2010 and December 2019 were enrolled in this retrospective study. These abovementioned patients were divided into two groups based on the Deauville criteria. The Kaplan–Meier method was used in survival analysis, and the log-rank method was adopted in comparison. Prognostic factor analysis was performed by the Cox regression model.

**Results:**

Positive post-ASCT [^18^F]F-FDG PET-CT was associated with lower progression-free survival (PFS) and overall survival (OS) (*p* = 0.001 and *p* = 0.022, respectively). Univariate analysis showed the post-ASCT PET-CT result was the only independent factor associated with PFS (*p* = 0.002). Both the number of previous treatments and post-ASCT PET-CT result had a different impact on OS (*p* = 0.040 and *p* = 0.028, respectively). Multivariate analysis showed the post-ASCT PET-CT result was the only independent factor associated with OS (*p* = 0.028). The results showed no significant change from the abovementioned results when DS < 3 was defined as the negative result. For patients who had a PET-CT scan within 3–6 months after ASCT, the negative PET-CT group had a better prognosis including PFS and OS (*p* = 0.009 and *p* = 0.025, respectively). However, among the patients receiving PET-CT within 3 months, the result was not statistically significant (*p* = 0.064 and *p* = 0.445, respectively).

**Conclusion:**

Collectively, we found that the post-ASCT [^18^F]F-FDG PET-CT was a strong indicator for PFS and OS, and a time window of 3–6 months was appropriate for post-ASCT [^18^F]F-FDG PET-CT. Trial registration number: ChiCTR2100042745.

## 1. Introduction

According to the statistics of GLOBOCAN 2018 produced by the International Agency for Research on Cancer, the number of new cases of Hodgkin's lymphoma (HL) and non-Hodgkin's lymphoma (NHL) is 79,990 and 509,590, respectively [1]. In China, there is an estimate of 52,000 deaths associated with lymphoma and myeloma, and the crude mortality rate was 3.83 per 100,000 in 2017 [2]. Hematopoietic stem cell transplantation (HSCT) plays a very important role in the treatment of lymphoma [3, 4]. Salvage chemotherapy followed by high-dose chemotherapy and rescue autologous stem cell transplantation (ASCT) remains the standard therapeutic regimen for relapsed/refractory lymphoma [5, 6]. 2-Deoxy-2-[^18^F]fluoro-D-glucose positron emission tomography/computed tomography ([^18^F]F-FDG PET-CT) has become an important tool to evaluate the prognosis of lymphoma since PET-CT is incorporated into the National-Cancer-Institute-sponsored international consensus response criteria for lymphoma guidelines in 2007 [7–9]. Prognostic assessment can help patients with lymphoma choose more promising treatment options in the early stage after ASCT. Patients with a good prognosis would not need to receive excessive treatment to reduce long-term toxicity. For lymphoma patients receiving ASCT, it has been shown that pretransplant PET-CT status is constantly and strongly associated with outcomes [9–13]. However, the prognostic value of post-ASCT [^18^F]F-FDG PET-CT still remains controversial. The Deauville score (DS) has been recommended as the preferred interpretation method for interim response evaluation [8, 14]. However, it still remains undetermined whether these Deauville criteria can be used in post-ASCT[^18^F]F-FDG PET-CT. Besides, given that posttherapy inflammatory changes may contribute to the “false-positive” PET-CT result, we hypothesized that a proper time window could help improve accuracy.

In the present study, we reported an updated analysis of using the DS to evaluate the prognostic value of early post-ASCT [^18^F]F-FDG PET-CT in patients with lymphoma.

## 2. Materials and Methods

### 2.1. Study Design

This study was approved by the medical ethics committee of the First Affiliated Hospital of Soochow University. Institutional databases were reviewed to identify patients with lymphoma who met the following inclusion criteria: all histologic types of NHL were allowed as well as HL, ASCT between January 2010 and December 2019 at the First Affiliated Hospital of Soochow University, and [^18^F]F-FDG PET/CT within 6 months after ASCT in our institution. The exclusion criteria were set as follows: patients who were lost to follow-up. Finally, 76 patients were included, and the data on these patients were analyzed.

Status at transplantation was determined according to the International Working Group criteria (IWGc) before ASCT [8]. Relapse or disease progression was defined according to the IWGc [7, 15].

This study was approved by the institutional review board of the First Affiliated Hospital of Soochow University. Trial registration number: ChiCTR2100042745. Because the trial was a retrospective study, written informed consent for this study was waived by the ethics committee, and no personal information was disclosed. This study was in accordance with the Declaration of Helsinki.

### 2.2. FDG-PET Imaging

The images were acquired on a GE Discovery STE16 PET/CT. All patients had blood glucose levels<11 mmol/L before injection. The dose of [^18^F]F-FDG was determined based on body weight, which was 4.07–5.18 MBq/kg (0.11–0.14 mCi/kg). Image acquisition for the whole-body PET scan started approximately 60 min after injection. Patients were imaged from the skull base to mid-thigh (approximately 2 min per bed position, with an average of 7–10 bed positions per scan). CT scans were obtained based on the correlative diagnostic CT images (3.5 mm/slice, 140 kV, 120 mA).

All serial scans were evaluated by two independent reviewers, who were blinded to clinical and radiologic data. The above mentioned patients were divided into two groups using the Deauville 5-point scale as indicated by Lugano's recommendations in lymphoma [8, 16]. DS 4 or 5 that could not be attributed to a physiologic or inflammatory cause was defined as the positive result, while DS < 4 was defined as the negative result. Considering using a fixed score for PET-positivity (i.e., DS 4) can leave outside less metabolically avid forms, and results by using DS < 3 a limit for negativity were also reported. In case of a discrepancy between the two observers, an independent panel of PET readers made the final decision.

### 2.3. Statistical Analysis

Overall survival (OS) was defined as the time from day 0 of ASCT to death or last follow-up for survivors. Progression-free survival (PFS) was defined as the time from day 0 of ASCT to the date of progression/relapse, death, or last follow-up without evidence of relapse or disease progression. The Kaplan–Meier method was used in survival analysis, and the log-rank method was adopted in comparison. Prognostic factor analysis was performed by the Cox regression model.

Characteristics considered for univariate analysis were age (<or ≥40 years), gender (male vs. female), type of lymphoma (B-NHL vs. T-NHL vs. HL), Ann Arbor stage (1–2 vs. 3–4), lactate dehydrogenase (LDH) (≤ vs.> upper laboratory limit (ULN)), previous lines of treatment (=1 vs. ≥2), Eastern Cooperative Oncology Group performance status (ECOG PS) (<2 vs. ≥2), extranodal lesions (<2 vs. ≥2), conditioning regimen (BEAM vs. BuCy vs. others), disease status at transplantation (complete remission and partial response vs. stable disease and progressive disease), and DS for PET-CT (DS < 4 vs. DS ≥ 4) after transplantation. Factors significantly associated with PFS or OS in univariate analysis were analyzed by multivariate analysis.

Statistical analyses were performed using the IBM SPSS Statistics (version 26.0). All tests were two sided, and *p* < 0.05 was considered statistically significant.

## 3. Results

### 3.1. Patient Characteristics

Between April 2010 and December 2019, 76 patients were enrolled in our present study according to the abovementioned inclusion criteria, including 51 males and 25 females. The median age of the cohort was 34 years (range 12–70). There were 62 NHL patients and 14 HL patients. There were 37 patients with B-cell NHL, including diffuse large B-cell lymphoma (*n* = 24), mantle cell lymphoma (*n* = 6), follicular cell lymphoma (*n* = 2), B lymphoblastic lymphoma (*n* = 2), Burkitt lymphoma (*n* = 1), small B-cell lymphoma (*n* = 1), and gray zone lymphoma (*n* = 1). Moreover, there were 25 patients with T-cell lymphoma, including anaplastic large cell (*n* = 10), and peripheral T-cell lymphoma not otherwise specified (*n* = 7), NK/T-cell lymphoma (*n* = 3), angioimmunoblastic T-cell lymphoma (*n* = 2), lymphoblastic lymphoma (*n* = 2), and subcutaneous panniculitis-like T-cell lymphoma (*n* = 1). A total of 37 patients received ASCT as consolidation therapy after first-line treatment, and 39 patients with recurrent or refractory lymphoma received ASCT as salvage consolidation therapy. There were 32 patients who underwent pretransplant PET. Besides, 43 patients underwent [^18^F]F-FDG PET-CT within 3 months after ASCT, and 33 patients had the scan within 3–6 months. The median follow-up time was 24 months (range 4–120).  Table 1 lists the characteristics of patients.

### 3.2. FDG PET-CT Results and Outcomes after ASCT

**Figure 1:**
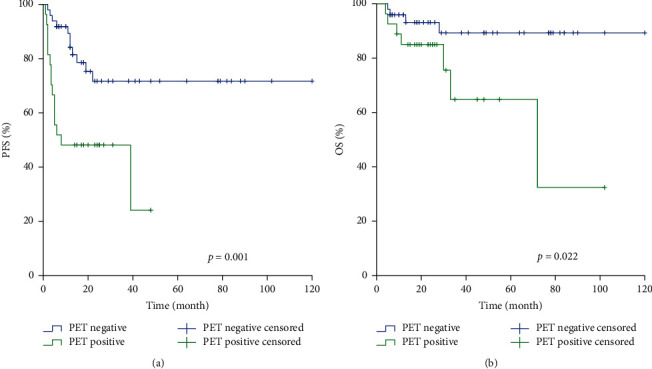
Kaplan–Meier analysis of PFS (a) and OS (b) for patients with [18F]F-FDG PET-CT findings after ASCT.

For all 76 patients with lymphoma, 26 patients (34.2%) relapsed/progressed at a median time of 5 months (95% CI, 4.64 to 11.47) after ASCT. Post-ASCT [^18^F]F-FDG PET-CT was positive for 27 of 76 patients (35.5%). Positive pre-ASCT [18F]F-FDG PET-CT was associated with lower PFS (*p* = 0.032) but not OS (*p* = 0.527). Positive post-ASCT [^18^F]F-FDG PET-CT was associated with lower PFS and OS (*p* = 0.001 and *p* = 0.022, respectively) (Figure 1(a), 1(b)). The 3-year PFS of the positive PET group and negative PET group was 72.3% and 48.1%, respectively. The 3-year OS of the abovementioned two groups was 89.7% and 64.8%, respectively.

### 3.3. Univariate and Multivariate Analyses

In the univariate analysis, the post-ASCT [^18^F]F-FDG PET-CT result was the only independent factor associated with PFS (*p* = 0.002; HR, 3.432; 95% CI, 1.585–7.526). Both the number of previous treatments and post-ASCT [^18^F]F-FDG PET-CT result were associated with OS (*p* = 0.040; HR, 4.981; 95% CI, 1.075–23.084 vs. *p* = 0.028; HR, 4.078; 95% CI, 1.161–14.320). Age, sex, type of lymphoma, Ann Arbor stage, ECOG PS, extranodal lesions, conditioning regimen, and status at transplantation had no impact on PFS and OS (Table 2). In the multivariate analysis, the post-ASCT [^18^F]F-FDG PET-CT result was the only independent factor associated with OS (*p* = 0.028; HR, 4.078; 95% CI, 1.161–14.320) (Table 3).

### 3.4. Timing of Post-ASCT [^18^F]F-FDG PET-CT and Outcomes

**Figure 2:**
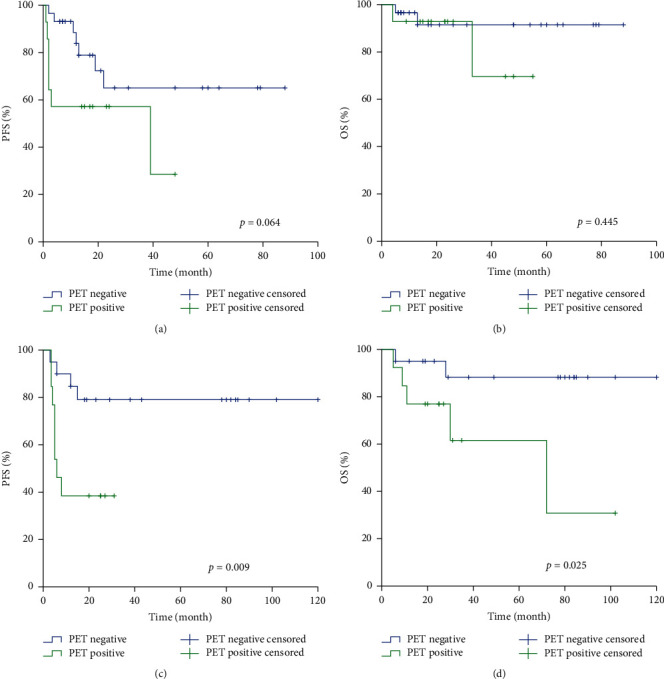
Kaplan–Meier analysis of PFS and OS for patients with [18F]F-FDG PET-CT findings within 3 months (a), (b) and within 3–6 months (c), (d) after ASCT.

For patients who had a PET-CT scan within 3–6 months after ASCT, the negative PET-CT group had a better prognosis, including PFS and OS (*p* = 0.009 and *p* = 0.025, respectively) (Figure 2(c), 2(d)). However, among the patients receiving post-ASCT [^18^F]F-FDG PET-CT within 3 months, there was no significant difference in PFS and OS between the positive PET-CT group and negative PET-CT group (*p* = 0.064 and *p* = 0.445, respectively) (Figures 2(a) and 2(b)).

### 3.5. Results by Using DS < 3 as the Limit for Negativity

The results showed no significant change from the abovementioned results when DS < 3 was defined as the negative result. Positive post-ASCT [18F]F-FDG PET-CT was associated with lower PFS and OS (*p* = 0.002 and *p* = 0.020, respectively). In the univariate analysis, the post-ASCT [18F]F-FDG PET-CT result was associated with both PFS and OS(*p* = 0.005; HR, 3.477; 95% CI, 1.456–8.303 vs. *p* = 0.036; HR, 5.205; 95% CI, 1.114–24.328). In the multivariate analysis, the post-ASCT [18F]F-FDG PET-CT result was the only independent factor associated with OS (*p* = 0.036; HR, 5.205; 95% CI, 1.114–24.328).

## 4. Discussion

Disease recurrence/progression is one of the main causes of HSCT failure and death in patients, especially those receiving ASCT. In the present retrospective study, we aimed to use DS to evaluate the prognostic value of post-ASCT [^18^F]F-FDG PET-CT in patients with lymphoma. Here, positive post-ASCT [^18^F]F-FDG PET-CT was associated with lower PFS and OS. In univariate and multivariate analyses, the post-ASCT [^18^F]F-FDG PET-CT result was an independent factor associated with both PFS and OS. We also found that, for patients who had a PET-CT scan within 3–6 months after ASCT, the negative PET-CT group had a better prognosis, including PFS and OS. However, an opposite conclusion was drawn among patients who had a PET-CT scan within 3 months after ASCT. This early evaluation could be crucially important because it is performed before the occurrence of relapse in most patients [17–19].

Several studies have investigated the prognostic value of pre-ASCT [^18^F]F-FDG PET-CT [20–28]. It has been confirmed that pre-ASCT [^18^F]F-FDG PET-CT can help identify lymphoma patients for treatment failure with ASCT. In this cohort, only 32/76 patients underwent pretransplant PET. Also, the result showed positive pre-ASCT [18F]F-FDG PET-CT was associated with lower PFS but not OS. This inconsistency may be attributed to the deficient number of cases. A few of studies have assessed the prognostic value of post-ASCT [^18^F]F-FDG PET-CT for lymphoma [29–34]. Most of these studies have confirmed the prognostic value of post-ASCT [^18^F]F-FDG PET-CT [29–31]. However, in Palmer's multivariate analysis, the post-ASCT [^18^F]F-FDG PET-CT scan cannot predict the outcome for patients undergoing ASCT [32]. In our retrospective study, we found that lymphoma patients with negative post-ASCT [^18^F]F-FDG PET-CT results had a better prognosis compared with the positive group, indicating that the post-ASCT [^18^F]F-FDG PET-CT result was an independent factor associated with both PFS and OS. Furthermore, in our univariate analysis, the number of previous treatments was an independent factor associated with OS but not PFS. However, in the multivariate analysis, the number of previous treatments was associated with neither PFS nor OS. Nevertheless, several previous studies have shown that patients receiving ASCT as a first-line consolidation treatment can have better outcomes compared with patients with relapsed/refractory lymphoma [35–37]. This discrepancy may be related to different PET criteria and different time windows for post-ASCT [^18^F]F-FDG PET-CT.

To minimize the frequency of posttherapy inflammatory changes, which potentially make the PET-CT result “false-positive,” PET should not be performed before at least 3 weeks after chemotherapy and preferably 8 to 12 weeks after the completion of radiotherapy [38]. However, the appropriate time window for post-ASCT [^18^F]F-FDG PET-CT remains largely undetermined. Several new agents have been reported to modulate tumor metabolism, glucose uptake, and inflammation in the tumor microenvironment, therefore potentially increasing the false-positive or false-negative [^18^F]F-FDG-PET results [39]. Although Ulaner et al. have reported that patients receiving ASCT rarely demonstrate FDG-avid lesions suggestive of disease without malignant pathology [40], in their research, the posttransplantation PET-CT for each patient is performed between 1 and 16 months after transplantation (median 6 months). In other previous studies, the time window for posttransplantation PET-CT is inconsistent, some even within 1 month [26, 41, 42]. In our current study, we tended to narrow down the time window to make the result more accurate. We separately studied the effects of PET-CT on PFS and OS within 3 months and within 3–6 months after ASCT. We found that PET-CT within 3–6 months, but not within 3 months, after ASCT was valuable in prognosis, suggesting that a time window of 3–6 months after ASCT was more appropriate for post-ASCT [^18^F]F-FDG PET-CT.

There are some limitations in the present study, such as the retrospective nature of this study, resulting in nonstandardized treatment and timing of follow-up examinations. Moreover, it would be better if we could describe the value of [^18^F]F-FDG PET-CT in more homogeneous cohorts of patients depending on the histologic type of lymphoma.

## 5. Conclusions

Taken together, we found that the positive post-ASCT [^18^F]F-FDG PET-CT result was associated with lower PFS and OS, and a time window of 3–6 months might be appropriate for post-ASCT [^18^F]F-FDG PET-CT. These findings provided valuable insights into the prognosis value of post-ASCT [^18^F]F-FDG PET-CT and might be used to guide the following treatment for patients with lymphoma.

## Figures and Tables

**Table 1:** Patient characteristics (*N* = 76).

Characteristics	No.
Sex	
Male	51
Female	25
Age (range)	34 (12–70)

Type of lymphoma	
HL	14
B-cell NHL	37
T-cell NHL	25
Median follow-up, month (range)	24 (4–120)

Number of previous treatments	
1	37
≥2	39

Ann Arbor stage	
I-II	17
III-IV	59

Extranodal lesions	
<2	49
≥2	27

LDH	
≤ULN	61
>ULN	15

ECOG PS	
<2	74
≥2	2

Conditioning regimen	
BEAM	56
BuCy	10
Others	10

Status at SCT	
CR + PR	59
SD + PD	17

Post-auto-SCT FDG PET-CT	
Negative (DS < 4)	49
Positive (DS = 4 or 5)	27

Timing of post-auto-SCT FDG PET-CT	
Within 3 months	43
Within 3–6 months	33

HL, Hodgkin's lymphoma; NHL, non-Hodgkin's lymphoma; SCT, stem cell transplantation; CR, complete response; PR, partial response; SD, stable disease; PD, progressive disease; ULN, upper laboratory limit; ECOG PS, Eastern Cooperative Oncology Group performance status; FDG, fluorodeoxyglucose; DS, Deauville score; PET, positron emission tomography; CT, computed tomography.

**Table 2:** Univariate Cox hazard analysis of risk factors for PFS and OS.

Variables	PFS		OS	
	HR (95% CI)	*p*	HR (95% CI)	*p*
Sex				
Male				
Female	0.702 (0.295–1.672)	0.413	0.437 (0.094–2.025)	0.29
Age				
≤40 years				
>40 years	0.653 (0.284–1.502)	0.315	3.254 (0.946–11.193)	0.061
Type of lymphoma				
HL		0.156		0.668
B	0.492 (0.175–1.385)	0.179	2.503 (0.308–20.358)	0.391
T	1.132 (0.418–3.065)	0.807	1.858 (0.193–17.882)	0.592
Ann Arbor stage				
I-II				
III-IV	1.366 (0.514–3.631)	0.532	31.09 (0.086–11310.060)	0.253
Extranodal lesions				
<2				
≥2	1.116(0.496–2.509)	0.791	1.448 (0.442–4.747)	0.541
LDH				
≤ULN				
>ULN	1.662 (0.698–3.961)	0.251	1.662 (0.440–6.285)	0.454
ECOG PS				
<2				
≥2	1.640 (0.221–12.166)	0.628	6.050 (0.725–50.467)	0.096
Conditioning regimen				
BEAM		0.344		0.818
BuCy	0.606 (0.141–2.611)	0.502	1.145 (0.136–9.665)	0.901
Others	1.820 (0.679–4.883)	0.234	1.654 (0.349–7.845)	0.526
Number of previous treatments				
1				
≥2	1.827 (0.825–4.046)	0.137	4.981 (1.075–23.084)	0.04^*∗*^
Status at SCT				
CR + PR				
SD + PD	1.235 (0.492–3.097)	0.653	0.950 (0.204–4.415)	0.948
Post-auto-SCT FDG PET-CT				
Negative (DS < 4)				
Positive (DS = 4 or 5)	3.432 (1.585–7.526)	0.002^*∗*^	4.078 (1.161–14.320)	0.028^*∗*^

^*∗*^ *p* < 0.05.

**Table 3:** Multivariate analysis of risks factors for OS.

Variables	OS	
	HR (95% CI)	*p* value
Number of previous treatments		
1		
≥2	—	0.097
Post-auto-SCT FDG PET-CT		
Negative (DS < 4)		
Positive (DS = 4 or 5)	4.078 (1.161–14.320)	0.028^*∗*^

^*∗*^ *p* < 0.05. DS, Deauville score; PET, positron emission tomography; CT, computed tomography.

## Data Availability

The underlying data supporting the results of our study can be found at http://www.chictr.org.cn/index.aspx.
